# What Matters Most: Improving Communication with Families of Periviable Infants

**DOI:** 10.3390/children13101369

**Published:** 2026-10-09

**Authors:** Kelsi Morgan, Claire A. Crawford, Shweta Parmekar, George Mandy, Karen E. Johnson

**Affiliations:** 1 Department of Pediatrics, Division of Hospice and Palliative Medicine, Northwestern University, Chicago, IL 60611, USA; 2 Lurie Children’s Hospital, McGaw Medical Center, Northwestern University, Chicago, IL 60611, USA; 3 Department of Pediatrics, Division of Palliative Care, Baylor College of Medicine, Houston, TX 77030, USA; 4 Division of Palliative Care, Texas Children’s Hospital, Houston, TX 77030, USA; 5 Department of Pediatrics, Division of Neonatology, Baylor College of Medicine, Houston, TX 77030, USA; 6 Division of Neonatology, Texas Children’s Hospital, Houston, TX 77030, USA

**Keywords:** communication, periviable infants, family-centered care

## Abstract

**Objective**: Little is known about the communication preferences of families of infants born in the periviable stage (20 weeks 0 days to 25 weeks 6 days) during the NICU course. This study aims to describe the communication preferences of parents of infants born in the periviable period. **Study Design**: Semi-structured interviews were conducted with 25 parents of periviable infants who were admitted to a 56-bed, level III NICU in the Southern United States from January 2023 to May 2024. Thematic analysis was performed. **Result**: Parents identified important preferences, including a desire for medical providers to give anticipatory guidance, to respect parents’ desire to maintain hope for their child, to be attuned to the emotional state of families and be empathetic in communication, and to value each individual family’s circumstances and experiences in the care of their child. **Conclusions**: Our study provides insight into the communication preferences of families with infants born in the periviable period.

## 1. Introduction

Over time, the gestational age at which infants are resuscitated has decreased, and survival rates of infants born in this periviable period, defined as 20 weeks 0 days to 25 weeks 6 days, has increased [1,2]. While survival rates have increased overall, infants born at these gestational ages still face significant morbidity and mortality, and surviving infants experience lengthy hospitalizations [2].

These NICU admissions can be stressful for families. It is well known that parents of infants in the NICU experience higher rates of anxiety than other similar parents, and some also experience post-traumatic stress [3]. Uncertainty about each baby’s clinical course likely contributes to this stress. Despite its importance, little is known about the quality of communication between families and healthcare providers in the NICU. A recent study described key factors valued by families and providers in communication in the NICU, including forming connections and building trust with families, supporting families in making decisions, and providing hope to families [4]. While studies have been conducted to improve the quality and frequency of communication between providers and families in the NICU [5], no study has looked at the unique communication needs of families of periviable infants after the birth of their infant.

While little is known about the communication needs of these families after delivery, several studies have focused on what families of periviable infants desire in a prenatal consultation. Recent data suggests that while neonatologists consistently share relevant clinical information in prenatal consults, few elicit family values as a part of the process [6]. Both prior to and after delivery, tailoring communication to each family’s needs is essential. This is especially true for families of babies born at the edge of viability [7].

While standardized tools do not exist for communication in the NICU with families of periviable infants, there are examples of analogous tools in other areas of pediatric medicine. Within the realm of pediatric oncology, a novel communication intervention called the “Day 100 Talk” was created in order to improve communication between families and healthcare providers during the first six months of pediatric cancer treatment [8]. This talk was created to reduce communication gaps between providers and families and was developed with input from families of children undergoing cancer treatment and the healthcare providers taking care of them. Families described the talks as helpful and worthwhile, and all families said they would recommend participating in the intervention to other families [9].

In this study, we aimed to create a similar intervention for parents of infants born in the periviable period. In this two-part study, our first aim was to characterize the communication preferences of families of periviable infants.

## 2. Methods

This study utilized qualitative methods to better understand the communication preferences of parents of infants born in the periviable period with healthcare providers in the NICU. The authors obtained IRB approval for this study.

Potentially eligible participants were families of infants admitted to a 56-bed, level III NICU in the Southern United States between January 2023 and May 2024. Included families had infants born between 22 weeks 0 days and 25 weeks 6 days. Additional inclusion criteria were having an infant who was inborn and family’s primary language being English or Spanish. Exclusion criteria were having an infant who died less than one year prior to the start of the study or if the infant was no longer in the birthing parent’s custody.

Families were contacted via phone beginning in September 2024, and informed consent was obtained prior to completing interviews. All families provided consent for interviews to be recorded. Semi-structured interviews were conducted in English or in Spanish via phone or in person from September to December 2024 by the first author, who speaks both English and Spanish. In cases where two parents from the same family participated, they were interviewed together. The interview guide contained questions aimed at better understanding families’ experiences with communication in the NICU and about their impressions of a proposed communication intervention in the NICU (Supplementary Materials File S1). The interview guide was based on the interview guide by Feraco et al. with adaptations for the NICU context [8].

Parents filled out a brief demographic survey at the end of their interviews which asked their age, highest education level, race, ethnicity, and preferred language. For families who did not fill out the demographic information sheet, the chart was reviewed for demographic information to be included when available. Interviews were audio recorded and transcribed. Transcriptions were verified against the audio recordings and personal identifiers were removed. Families received a $25 gift card for participation.

CAC, who has expertise in qualitative methodology, provided guidance regarding the structure of the study and qualitative analysis of transcripts. An inductive, phenomenological design was used to analyze the lived experiences of parents who shared the phenomenon of caring for a periviable infant in the NICU [10]. Two of the researchers (KMM and KEJ) acted as coders and met with CAC regularly throughout the coding process. KMM and KEJ followed an inductive process to code the transcript text with open coding, independently generating and recording initial codes in Atlas.ti (web version 9.3.0). They met to reconcile and develop shared codes after each of the first 5 interviews. After general consensus was reached to create a common codebook, the coders proceeded to independently code the remaining transcripts. Once they met to finalize and agree on the codebook, KMM and KEJ searched for shared meaning within the codes and placed them into larger categories that became the subthemes. The most salient shared concepts from the subthemes became the final larger themes. CAC assisted in conceptualizing the subthemes and their development into themes resulting from the codebook analysis.

## 3. Results

### 3.1. Parent and Patient Demographics

A total of 62 families of 67 infants (five sets of twins) were identified as having infants in the period of interest, of which 55 met eligibility criteria for this study. Twenty-eight families did not respond to attempts to make contact via phone (defined as two unanswered phone calls) or stopped responding to phone calls after initial contact was made. Seven families declined to participate. A total of 25 individuals as part of 20 families participated in the study (20 mothers and five fathers). Almost half of parents identified as Hispanic and one-third of parents identified as Black. One-third of represented infants were born between 22 weeks 0 days and 23 weeks 6 days, and 86% of infants survived to discharge. Additional demographic information of the participating parents and their children is provided in Table 1.

### 3.2. Thematic Results

**Figure 1:**
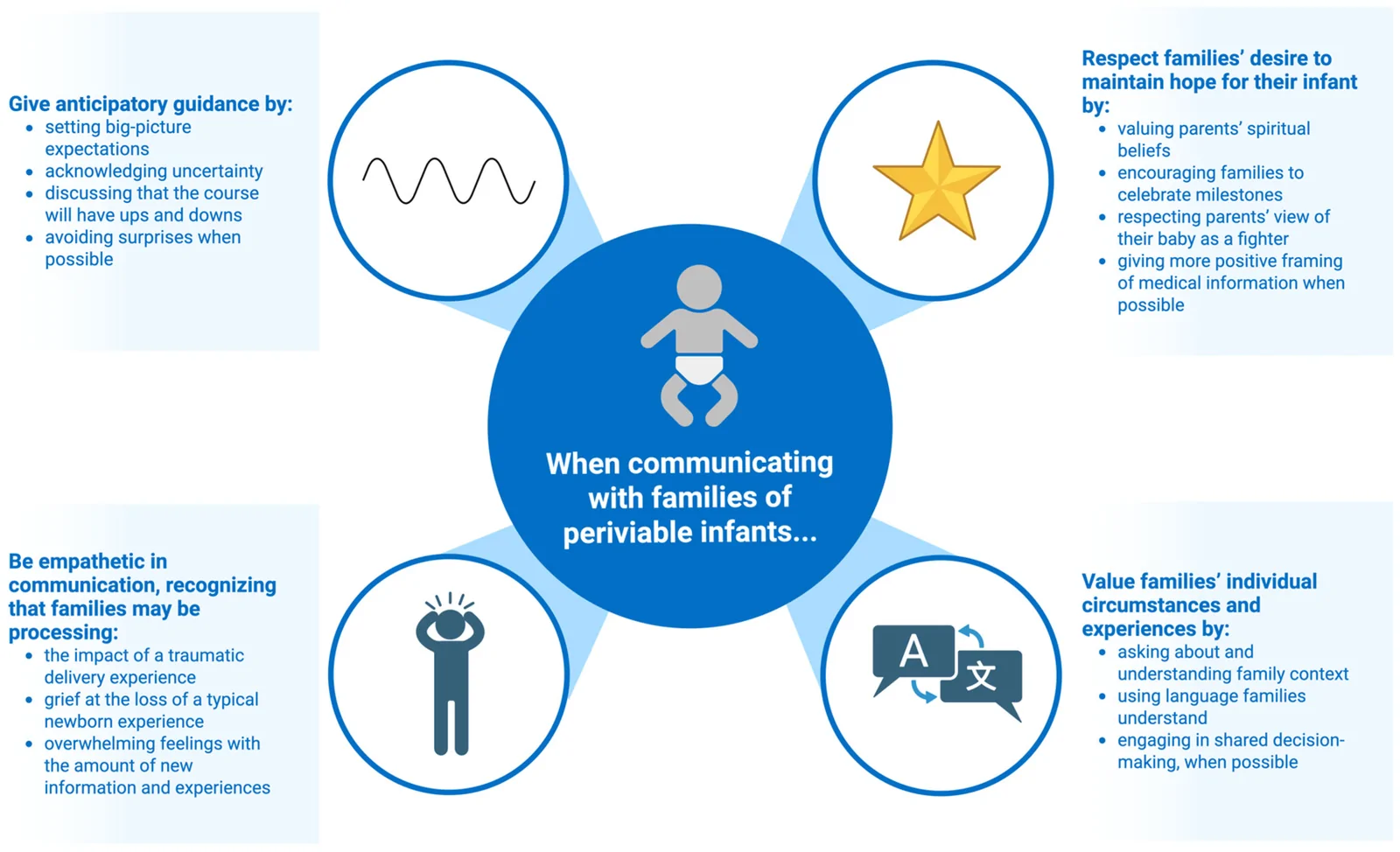
Communication preferences of parents of infants born in the periviable period.

Four major themes were identified: importance of anticipatory guidance, importance of hope, importance of empathetic communication, and the importance of valuing families’ individual circumstances and experiences (Figure 1). Subthemes were identified in each category.

### 3.3. Theme 1: Importance of Anticipatory Guidance

#### 3.3.1. Subtheme 1: Set Big-Picture Expectations

Families described a desire for their healthcare teams to discuss overarching expectations early in their child’s life when possible. In the words of one parent, “I know that things are kind of unpredictable, but I think proactively maybe talking to the families about, based on that baby’s case, what the different—maybe not the different outcomes, but the different timelines can be. Because at first, we had no clue, like, are we going to be here a year? Are we going to be a few weeks?” [Interview 17] Having a big-picture discussion that goes beyond the day-to-day updates can be helpful as parents process the early arrival of their infant.

#### 3.3.2. Subtheme 2: Acknowledge Uncertainty

While parents wanted to participate in a discussion of big-picture expectations, they also acknowledged that there is a great deal of uncertainty in every baby’s course. Several parents described a desire for providers to acknowledge this uncertainty explicitly. One mother stated “[healthcare providers ought] not give parents hope that their babies are going to be okay. But just—the baby next to him coded and passed within a week of the first time he coded, shortly after [our baby] had coded. And so I just—knowing that they go through that honeymoon phase and that things could go a completely different way, I think, is really important for parents to hear.” [Interview 5] Parents emphasized the importance of continuing to acknowledge this uncertainty throughout the hospital course. When discussing the possibility of long-term oxygen use, one parent described her experience as follows:

“Because mine, thank God, after about a month and a half, they took [the oxygen] off. But I met a friend, a lady here, her baby was already five months old and was still on it. So it’s important that they tell you the truth about what is not known. Everything depends on the baby. Yes, the nurses always said it, they always said—I asked them, ‘When are they going to take this off?’ [The nurses said], ‘No, I can’t tell you, everything depends on him. Everything depends on him.’”

[Interview 7]

#### 3.3.3. Subtheme 3: Discuss That the Course Will Have Ups and Downs

Several parents discussed their experience in the NICU as a journey with many ups and downs. Multiple families described this phenomenon as a rollercoaster: “It’s gonna be like a rollercoaster here, you know, baby starts doing very well and then, all of the sudden, sometimes the baby’s pretty sick…so it did happen that it was, that she had some good days, and then she had some bad days.” [Interview 14] From another parent: “It’s like a rollercoaster. It goes up and down.” [Interview 11] Parents described their desire for providers to engage in conversation with them about this phenomenon, using it to contextualize the journey of their premature infants: “reiterate that so much—like, he’s doing great right now, don’t get your hopes up. Don’t get your hopes up.” [Interview 5] By having a conversation about these ups and downs, providers may be able to normalize this phenomenon for families, which may act as a form of anticipatory guidance.

#### 3.3.4. Subtheme 4: Avoid Surprises When Possible

While parents were not uniform in the amount of information they desired from medical teams, one consistent request that emerged was parental desire for medical teams to avoid surprises when possible. Even regarding neutral or positive shifts, parents described wanting to know about anticipated changes. As one mother said, “I know we, throughout the process, we’ve walked in and they’re like, oh, there’s this new…she went from the isolette to this to that. But maybe [the medical team could be] explaining the process ahead of time of what can happen…I think we were surprised on some things.” [Interview 3] One mother described wanting more information as a form of preparation in the face of uncertainty: “I like information. I don’t like feeling like I’m going to have to make a big decision and it just, like, be a surprise.” [Interview 13]

### 3.4. Theme 2: Importance of Hope

#### 3.4.1. Subtheme 1: Value Parental Spiritual Beliefs

While several parents described the involvement of spiritual care and chaplaincy services in critical periods, such as during a code event, some families would have liked spiritual support earlier in their child’s NICU journey. One mother said: “So I think for people who are…Christians and are Muslim, they would really appreciate if a clergy would be involved from the beginning until the end. Because it becomes part of the process of every situation that you’re going through. That would have really helped me.” [Interview 8] Parents also described the importance of faith as they faced challenges in the NICU. One parent described that their child’s outcome was “in God’s hands. I just need to pray about it and leave it there.” [Interview 13] For some parents, faith was a source of strength in the face of uncertainty.

#### 3.4.2. Subtheme 2: Encourage Parents to Celebrate Milestones

Despite the difficulties of a NICU stay, families were clear in their desire to celebrate every small achievement their babies made. As one parent stated, “But also it’s exciting, you know, just to know what will be new. I mean, we were happy every time that we got a little charm from the necklace from the NICU. And every time that we got the opportunity to do little things with the baby—change the diapers, I would start doing the dressing changes and stuff like that…the stimulation with the oral care. That was really awesome. It’s little things like that.” [Interview 10]

#### 3.4.3. Subtheme 3: Respect Parents’ View of Baby as a Fighter

Several parents emphasized that, though their infants were born tiny, they were strong. The characterization of their baby as a “fighter” was important to many families. One parent reflected on the early days of their son’s life: “And he was an active little baby, you know. That was what really gave us hope—that for a baby his size, he was really moving.” [Interview 8] Another parent described even a potentially scary medical event—an unplanned extubation—as evidence of their baby’s fight and strength: “You’re going to see a lot of up and down, but these babies are very strong. They’re very strong. I noticed that they’re strong—very, very strong—because she would try to pull her vent tube out.” [Interview 14] Seeing their babies as strong and spirited was important to many families.

#### 3.4.4. Subtheme 4: Give Positive Framing of Medical Information

Several families expressed sadness that some healthcare providers did not seem to join them in having hope for their babies’ survival. One mother described persistent negative framing of news from healthcare providers: “So, yeah, because I honestly believe that in the NICU, we hear so much bad of, well, this could happen, that could happen, just more of the bad stuff versus the good things that are happening. So I think probably hearing a bit more positivity around how our babies are doing would be more uplifting for the parents from the kind of emotional standpoint.” [Interview 9] Families want to feel that the medical teams are rooting for their babies alongside them. One mother described how this perceived lack of hope impacted her: “But the only thing that I would have liked—and that happened through my NICU stay with the doctors, many of the doctors, not just with that neonatologist before I delivered the baby—it wasn’t always very…I didn’t feel like they were very hopeful. Like the way they gave me the information, it was always very factual. Like, okay, this might happen. Or we might need to do this. Da-da-da, like that. But at the end of the day, these were my children.” [Interview 19]

### 3.5. Theme 3: Importance of Empathetic Communication

#### 3.5.1. Subtheme 1: Parents May Be Processing the Impact of a Traumatic Delivery Experience

While parents of infants born in the periviable period are processing the birth of their infant, they are also processing a delivery experience that has been many months earlier than anticipated, and in many cases, sudden and traumatic. One mother recalled “Yeah, it just went so fast. It just happened, and I was just like…I just woke up, and then they had told me that she went to the NICU.” [Interview 4] Echoing the experience of a rapid delivery followed by serious complications, one mother stated “I just went into labor and everything happened so fast. And apparently, my baby was breech, and I had to get an emergency c-section, which caused a serious infection that went on for days, and it was a horrible experience, which has scared me for life.” [Interview 12] Because delivery was often quick, many families described being unable to receive a prenatal consult or remembered very little of what was shared. As one parent stated, “It was so quick that I didn’t talk to no NICU…the only person I talked to was the midwife and the nurses that were there with me. And then when everything started, me having contractions that night, and then everything…it happened so quick that they took me to the c-section right after that. I hadn’t, they didn’t get to explain anything cause everything went so quick.” [Interview 14]

#### 3.5.2. Subtheme 2: Parents May Be Experiencing Grief of the Loss of a Normal Newborn Experience

Because delivery of an infant in the periviable period is unexpected, many parents expressed grief at the loss of a typical newborn experience. Experiences many parents of term infants take for granted, such as sharing a room with their infant or being able to dress their baby in a particular outfit, were sources of grief. As one mother described, “I was really sad because she wasn’t in the room with me, and I had to leave her…and then I couldn’t touch her.” [Interview 4] She later went on to say “So I didn’t know that she couldn’t wear clothes. I didn’t know that she couldn’t wear clothes yet, and they were like, ‘Oh, well, maybe we’ll wait a couple months.’ So don’t, don’t, like, you know? So those conversations were kinda hard.” [Interview 4]

#### 3.5.3. Subtheme 3: Parents May Be Feeling Overwhelmed with Information and Experiences

Beyond processing the trauma of an unexpected delivery and grief at the loss of a typical experience, parents may also be feeling overwhelmed and sometimes shocked at the sheer volume of information they are receiving from providers in the early days of their infant’s life. One mother described her experience with trying to process all of this at once: “Everything was very confusing…there was a lot of information that they would give me every time I go into the room. I have all the doctors like, oh so with (baby’s name), their lungs, this happened with the lungs, and then his vent, and he needs to have this, this new machine for that. And we have to do the procedure, and the lines…and there was just a lot of things.” [Interview 19] While it is essential to keep parents well-informed, it is also essential to keep in mind the multiple emotions and experiences families may be processing during this early newborn period.

### 3.6. Theme 4: Value Individual Circumstances and Experiences

#### 3.6.1. Subtheme 1: Ask About and Understand Family Context

Because each parent may have unique communication preferences, parents advocated for having direct conversations about how families prefer to receive information. One parent described how this could happen: “I think that also another good idea would be at the beginning, or once I guess baby is more stable, I don’t know if it’s the social worker, or any other person that can ask that type of questions, like what type of person, what type of family are you? How do you handle depression? How do you want to hear, or how do you want to talk about things that are kind of bad? So that you guys can kind of get to know the family, the person that you’re going to talk to.” [Interview 10] In addition, families described how previous experiences shape how they prefer to receive information from healthcare providers. For one mother who had a previous infant in the NICU, it was important for the medical team to understand her family’s prior experiences: “They asked me quite a few questions about my first experience at [the hospital] with [my other child]. They really got to kind of understand my capacity of understanding whenever it comes to the medical NICU world and things like that. So I think that by them really taking the time to understand my previous experience, and then also the difficulties that we had with [my other child] really did allow for them to be a bit more candid with me and be a bit more open with me as well.” [Interview 9] By taking time to understand a particular family’s context and communication preferences early in the hospitalization, this can help with communication throughout the NICU stay.

#### 3.6.2. Subtheme 2: Use Language Families Understand

Several families discussed the importance of communicating in language each parent understands. This included the importance of using translators for medical information when needed and avoiding using jargon. One mother described her frustration: “I understand that for you as a doctor, it’s easier, faster, to cut the information between you guys as a medical team using the abbreviations. But for me as a parent, I’m not in the medical field, and I’m not even an English native speaker. It’s like, what? It makes the experience, I don’t want to say worse, because it’s already very bad, but it’s just scarier. Because it’s like maybe he’s telling me something really bad is happening, and I’m not understanding the severity of what he’s telling me because I’m not even understanding what that abbreviation is. Or maybe it’s something very simple, but I’m making it worse in my head because I don’t know what he’s talking about.” [Interview 19] Using unclear language can increase parental anxiety, even in the face of news that would otherwise not be anxiety-inducing.

#### 3.6.3. Subtheme 3: Engage in Shared Decision-Making, When Possible

Throughout the NICU stay, many parents described the importance of their role in the care team. Families described that they knew their child in ways the medical team did not. This became important in times of disagreement with the medical team. One parent described a conflict surrounding a trial of extubation: “We had a very serious conversation with the medical team and director because they wanted to extubate him. And I did not feel that he was ready for it. Because they, they thought it was—it was based on studies. This is what the doctor told me. Based on studies, this is the best time that we need to extubate him. And I was like, look, according to what your team, the previous team had said, they were wanting to wait to do the extubation on him until he was a certain weight. He is not gaining weight. He’s having issues tolerating feeds. We want to make the changes, but I want to make sure that whatever changes you make are also coming from the director. And the director came in, and he explained that, based on studies—and I was like, he’s not a study.” [Interview 15] Parents emphasized the importance of providers seeing their children as individuals. Another mother described a conversation with a medical provider as follows: “But I did, I had a talk with one of the residents, and I did keep repeating to him, like, hey, I understand that you guys don’t have a lot of time. Your time is very limited for many patients. But at the end of the day, you’re not talking to me about a… I’m not in a—how can I say it? Like in a medical congress. Like you’re not telling me about possibilities, probabilities, the percentages according to studies, according to this study in this paper. You’re talking about my kids. Remember that.” [Interview 19]

## 4. Discussion

In this qualitative study, we were able to characterize the communication preferences of families of infants born extremely prematurely, between 22 weeks 0 days and 25 weeks 6 days gestation. This study is novel in examining this particular population within this defined time frame. While attention has been given to communication preferences of families of periviable infants prior to delivery [11], no study has looked at communication preferences of this population during their early NICU course. Moreover, studies have looked at NICU communication preferences of parents generally, or of parents of late preterm infants, but no study has looked at parents of periviable infants specifically [12,13]. While helpful guides have been created outlining things to consider when communicating with this population [7], our study is the first to focus on the voices of parents themselves.

In interviews, families described their desire for anticipatory guidance, including communication of expected trajectories, acknowledgment of uncertainty, and emphasis on the ups-and-downs of a NICU course. Previous studies have identified the importance of acknowledging uncertainty of outcomes of extremely preterm infants in prenatal consults [11]. Even after birth, families desire that this uncertainty be explicitly acknowledged by providers.

While families were clear that there would be challenges throughout the NICU course, they wanted to feel that their providers were hoping for the best for their infants alongside them. Parents in this study echoed previous findings that parents wished providers would be more hopeful, rather than persistently negative, in their communication [12]. While it can be challenging for physicians to use positive framing in the face of prognostic uncertainty, hoping alongside families requires an understanding that families can hope for more than one thing at once [14]. Even in the face of life-changing news, it is part of the physician’s job to “help illuminate the hopes that remain” [14]. In practice, this may sound like telling a family who had just received bad news ‘I wish this news had been different,’ and following up any subsequent discussion of hopes for miracles or cures with ‘Do you mind telling me what else you might be hoping for?’ [14].

A notable thread in parents’ preferences was their desire to celebrate milestones. This is related to their desire to be included in their infant’s care—to have opportunities to feel like they were actively caring for their child as parents. A recent qualitative thematic synthesis about family-centered care in the NICU also found that parents wanted to be included in the daily care of their infants, including things like diaper changes and bathing [15]. Because families of periviable infants in particular may be hesitant to participate in cares given the extremely preterm state of their infants, it may benefit NICUs to have specific policies in place detailing ways to ensure parents are included. This may help families establish a sense of normalcy as parents even in the setting of the NICU.

Families also wanted medical providers to be empathetic in their communication. This is consistent with findings from previous research on both antenatal counseling and experiences of families generally in the NICU [16]. While many NICU courses are unexpected, a striking theme that emerged from our interviews was the degree to which many families felt overwhelmed by the early birth of their child resulting in difficulty remembering what they had been told or in processing new information. This phenomenon has been identified before: a previous qualitative study analyzing the experiences of parents meeting their very preterm babies for the first time identified that many families had difficulty recalling details around the birth of their child [17]. It would benefit providers to remember that many parents may not recall details from the prenatal consult and may need this information reiterated after delivery. Visual aids may help with recall. From a guidance standpoint, a recent review of periviable counseling practices advocated for a trauma-informed approach [18]. Approaching communication with families throughout the entire NICU course with a trauma-informed lens may be helpful.

While many families are processing similar emotions, our study found that families wanted their unique experiences to be valued. Janvier et al.’s paper on communication with parents about withdrawing or withholding life-sustaining interventions in the NICU advocates for assessing parents’ stories, concerns, and needs during serious discussions in the NICU [19]. The paper provides a helpful series of questions aimed at identifying key aspects of parents’ perspectives and preferences. Of note, in our study, families advocated for communication that incorporates many core principles of palliative care. Families wanted their infant’s doctors to ask questions about their stories, their communication preferences, and their values and hopes near the beginning of the NICU admission, rather than at a moment of decision for their infants. Getting to know families’ stories and preferences early in the NICU course may help teams communicate more effectively should challenges arise later in the hospitalization. In line with this preference to have individualized care, several families emphasized the importance of medical teams seeing their infant as more than just a number or a diagnosis. Parents wanted their views and preferences to be valued in decision-making, even when their preferences conflicted with what statistics would suggest. Being able to hear parents out in these moments requires humility, acknowledging that “the information that seems cutting-edge today may not appear accurate tomorrow” [20].

### Strengths, Limitations and Future Directions

Our study has several strengths. We were able to speak with many families about their experiences, and our interview population was diverse in age, race, ethnicity and experience. We were able to speak with families of all target gestational ages (22–25 weeks), and we were able to include perspectives of families with both surviving and deceased infants.

Our study also faced limitations. First, we interviewed parents after the discharge of their infants from the NICU (or after the death of their infant), so there may be bias in how families recalled their experiences. In addition, while we were able to speak with some fathers, the majority of participants were mothers. Likewise, while we were able to speak with two exclusively Spanish-speaking parents, we did not have robust representation of families who spoke a primary language other than English. There were also many families who did not respond to or declined a request for an interview. It is possible that these families differed from those who participated in a meaningful way, which could shape the results. Additional studies are needed to better understand the perspectives of non-birthing parents and families who prefer to communicate in a language other than English.

## 5. Conclusions

In summary, families of extremely preterm infants have communication preferences that are generally in line with those of other parents in the NICU. Palliative care teams prioritize many of families’ preferred aspects of communication, including eliciting goals and values early, acknowledging the infant as a person rather than just a patient, and consistently being empathetic. While this study did not directly examine families’ views of palliative care involvement, the study’s themes suggest that it may be helpful for NICU teams to consider involving palliative care teams early in the hospital course for these families. Palliative care clinicians are specifically trained in many of the communication preferences reflected in this study’s themes [21]. Alternatively, teams may also consider incorporating these aspects of communication in their care of these patients. Future work should focus on standardized ways to incorporate parents’ communication preferences into the NICU stay. With this goal in mind, our team is currently piloting a series of communication tools in the NICU based on the themes from this study.

## Figures and Tables

**Table 1:** Demographics of interviewed parents.

**Age**	**n**	**%**
≤25 years old	4	16
26–34 years old	7	28
≥35 years old	12	48
Information unavailable	2	8
**Gender**		
Male	5	20
Female	20	80
**Race/ethnicity**		
Hispanic/Latinx	12	48
Black	8	32
White	15	60
Multiracial	1	4
Information unavailable	1	4
**Highest education level**		
Some high school	1	4
High school diploma	4	16
Some college	3	12
Associates degree	1	4
Bachelor’s degree	9	36
Master’s or doctoral degree	2	8
Information unavailable	5	20
**Language of interview**		
English	23	92
Spanish	2	8
**Child’s gestational age at birth**		
22w0d–22w6d	3	14
23w0d–23w6d	4	19
24w0d–24w6d	7	33
25w0d–25w6d	7	33
**Child’s survival to discharge**		
Yes	18	86
No	3	14

Note: incomplete demographic information was collected for four parents.

## Data Availability

The dataset is not publicly available because interviews contain patient and parent identifiers.

## Supplementary Materials

The following supporting information can be downloaded at https://www.mdpi.com/article/10.3390/children13101369/s1: File S1: Guide used for parent interviews.
