# Supplementary material for: What Matters Most: Improving Communication with Families of Periviable Infants
Source: Children (Basel). 2026 Oct 9;13(10):1369. doi: 10.3390/children13101369 (PMC13703882; doi:10.3390/children13101369)
Supplement: Supplementary file 1 [file children-13-01369-s001.zip › children-4588822-supplementary.pdf]

Supplement File S1: Guide used for parent interviews.

Interview guide with families of infants born between 22-25 weeks gestation

Introduction:

Thank you so much for agreeing to talk with me today as a part of this research study. The goal of this study is to improve communication between healthcare providers and families of infants who are born very prematurely, between 22-25 weeks gestation. In order to do that, we are trying to better understand the experiences of families who have had infants born during this period. During our conversation, I'll ask you some questions about your experience in the NICU. This interview will take about 45 minutes. If I ask any question that you'd rather not answer, please let me know, and you do not need to answer that question. If you'd like to stop talking, please let me know, and we can stop the interview. It's also okay if you don't remember the answer to a question I ask. I'm recording this interview so that I don't miss anything you share. At the conclusion of this interview, you will receive a gift card as a token of appreciation for your time. Do you have any questions before we get started?

Prenatal consult

1. Did a doctor from the NICU come and talk with you before your baby was born about what to expect at delivery?
    a. What do you remember about that conversation?
    b. Was there any information you wish you would've gotten at that time?
    c. Was there any information you wish you would not have gotten at that time?

Delivery experience:

1. Tell me about the birth of __________.

First week in the NICU:

1. What do you remember about __________'s first few days in the NICU?
2. Do you remember the first time you got an update from the medical team about your baby? Can you tell me about that time?
3. Did you have any family meetings with your medical team during the first week?

4. Most babies get a head ultrasound in the first week of life. Do you remember a conversation about that head ultrasound?

First month in the NICU – General communication:

1. During your baby's first month in the NICU, how did you feel communication was between you and your baby's medical team?

2. What made it easy to ask questions of your baby's medical team?

3. What made it difficult to ask questions of your baby's medical team?

    a. Some families say that it was easy to ask questions when the team rounded, especially when teams were clear with their communication. Alternatively, some families say it was difficult when they didn't understand some of the medical words or they weren't able to be at the bedside when doctors were there. Did you ever have these experiences?

4. Can you tell me about a time in the first month of your baby's life that you had a serious conversation with your baby's medical team?

5. Was there anything you wish your medical team had understood about you or your family during your baby's time in the NICU, but didn't?

Description of intervention:

Thank you so much for answering those questions and for sharing your experience with me. We are hoping to create two planned conversations that would take place between families of infants born at 22-25 weeks and their medical teams in order to have "big picture" conversations about how their babies are doing. The idea is to have the first conversation when babies are about seven days old, and the second when babies are about one month old. The goal would be to have conversations that would not focus on the day-to-day updates, but would allow everyone to step back and talk about how the baby is doing, and what might be expected in the future.

1. What are your thoughts about these conversations?

    a. How might they be helpful?

    b. What might be the downsides of them?

2. Who do you think should be at these talks?

3. What do you think would be important to discuss during the talks?

Closing:

Thank you so much for taking the time to talk with me. Here is your gift card. Are there any concerns or questions you have about the conversation we've had today?
